# Whole-Genome Sequencing Uncovers Metabolic and Immune System Variations in Propionibacterium freudenreichii Isolates

**DOI:** 10.32607/actanaturae.27764

**Published:** 2025

**Authors:** I. D. Antipenko, S. A. Venedyukhina, N. P. Sorokina, I. V. Kucherenko, T. S. Smirnova, G. N. Rogov, M. Yu. Shkurnikov

**Affiliations:** Laboratory for Research on Molecular Mechanisms of Longevity, Department of Biology and Biotechnology, HSE University, Moscow, 101000 Russia; All-Russian Research Institute of Butter and Cheese Making, Branch of the Gorbatov Federal Research Center for Food Systems, Uglich, 109316 Russia

**Keywords:** Propionibacterium, whole-genome sequencing, metabolism, CRISPR-Cas, bacteriophages

## Abstract

Propionibacterium freudenreichii plays a crucial role in the production of Swiss-type cheeses; however, genomic variability among strains, which affects their technological traits, remains insufficiently explored. In this study, whole-genome sequencing and comparative analysis were performed on five industrial P. freudenreichii strains. Despite their overall high genomic similarity, the strains proved different in gas production and substrate metabolism. Phylogenetic analysis revealed a close relationship between strain FNCPS 828 and P. freudenreichii subsp. shermanii (z-score = 0.99948), with the latter being unable to reduce nitrates but being able to metabolize lactose. The narG gene encoding the nitrate reductase alpha subunit was detected in only one of the five analyzed strains - FNCPS 828 - and in 39% of previously described P. freudenreichii genomes, suggesting its potential as a marker of nitrate-reducing capability. Analysis of 112 genomes showed that the I-G CRISPR-Cas system was present in more than 90% of the strains, whereas the type I-E system was found in approximately 25%. All the five study strains harbored the type I-G system; strain FNCPS 3 additionally contained a complete type I-E system with the highest number of CRISPR spacers, some of which matched previously published bacteriophage sequences. The most prevalent anti-phage defense systems included RM I, RM IV, AbiE, PD-T4-6, HEC-06, and ietAS. These findings highlight the genetic diversity of P. freudenreichii strains, which is of great importance in their industrial applications. The identification of narG as a potential marker of nitrate-reducing activity, along with detailed mapping of CRISPR- Cas systems, boosts opportunities for the rational selection and engineering of starter cultures with tailored metabolic properties and increased resistance to bacteriophages.

## INTRODUCTION

Members of the genus Propionibacterium play an important role in the food industry. In particular, Propionibacterium freudenreichii strains are widely used in the ripening of Swiss-type cheeses [1]. The key metabolic pathway in P. freudenreichii is the Wood-Werkmann cycle, where lactate is first converted to pyruvate and then metabolized: one portion is converted to propionate that gives the cheese its characteristic flavor, and the other portion is converted to acetate and carbon dioxide that form the characteristic “eyes” [2].

Each P. freudenreichii strain is characterized by a unique set of enzymes that underlies the specific features of its metabolic activity [3], which affects fermented carbohydrates and provides the taste of the final product [4]. Furthermore, these bacteria synthesize vitamins B9 and B12, conjugated linoleic acid, trehalose, bacteriocins, and organic acids and exhibit probiotic properties [5].

Bacteriophage contamination is a serious problem for the dairy industry, because it can result in fermentation failure and product defects. Bacteriophages are detected in approximately half of Swiss-type cheeses at a concentration of at least 105 PFU/g; they multiply as propionic acid bacteria grow in a warm chamber during cheese ripening [6]. Given the key role of P. freudenreichii in shaping the organoleptic characteristics of cheeses, investigation of their immune defense systems is of great practical importance for identifying phage-resistant strains and minimizing the risk of process failures during the ripening stage [7].

Despite the industrial significance of P. freudenreichii, genomic characterization of industrial strains of this species is very limited. Whole-genome sequencing identifies interstrain variations and reveals links between genotype and technological properties, including metabolism, stress resistance, and defense systems [4].

In this study, we present the results of whole-genome sequencing of five P. freudenreichii strains used in the dairy industry and comprehensive genomic characterization of these strains, focusing on their metabolic characteristics, defense mechanisms, and functional gene variability

## EXPERIMENTAL

**Strains and culture conditions**

In this study, we used five P. freudenreichii strains: FNCPS 2 (GCA_044990475.1), FNCPS 3 (GCA_044990455.1), FNCPS 4 (GCA_044990515.1), FNCPS 6 (GCA_044990495.1), and FNCPS 828 (GCA_044990435.1) received from the collection of the All-Russian Research Institute of Butter and Cheese Making of the Dairy Industry (VNIIMS, a branch of the Gorbatov Federal Research Center for Food Systems of the Russian Academy of Sciences). Strains FNCPS 2 and FNCPS 3 were isolated from raw milk samples, and the others were isolated from cheese samples. All strains were isolated from dairy products manufactured in Altai Krai, Russia.

Propionibacterium bacteria were cultivated in a liquid culture medium containing peptone (10 g), yeast extract (10 g), cobalt chloride (0.01 g), potassium monobasic phosphate (1 g), and 20 cm³ of 40% lactic acid. These components were dissolved in 1 L of distilled water, the pH was adjusted to 7.1 ± 0.1, and the mixture was then poured into test tubes and sterilized at 121 ± 2°C for 15 min. The same medium was used to study the gas-producing activity of P. freudenreichii strains.

The effect of milk protein proteolysis products on gas production by propionibacterium was studied using the same culture medium. However, the components were added to pancreatin-hydrolyzed skim milk diluted with distilled water at a ratio of 1 : 2.

To produce propionibacterium cultures, the culture medium was inoculated with 1% of the inoculum and incubated in a thermostat at 30 ± 1°C for 72 h.

**Phenotypic characterization of the strains**

The rate of gas production and the volume of released gas were measured during culturing in graduated Dunbar tubes with a 1% inoculum dose at 30 ± 1°C. The volume of released gas was measured daily for 15 days. The rate of gas production was calculated as the maximum gas volume divided by the number of culture days.

The effect of temperature on the gas-producing activity of the cultures was assessed by culturing cells in graduated Dunbar tubes with a 1% inoculum dose at 18 ± 1°C, 22 ± 1°C, and 30 ± 1°C. The gas volume was measured daily for 15 days.

Anaerobic bacteria were identified by measurements of the biochemical activity of the cultures using the API 20A test system (bioMérieux, France) according to the manufacturer’s instructions. Test strip results were analyzed using the APIWEB online database (bioMérieux).

**Bacterial genome sequencing and assembly**

DNA for genome sequencing was isolated using an ExtractDNA Blood and Cells kit (Eurogen, Russia) according to the manufacturer’s instructions. DNA libraries were prepared using the MGIEasy Fast FS DNA Library Prep Set V2.0 (Cat. No. 940-001196-00, MGI, China) according to the manufacturer’s protocol. Library quality was assessed using a Qubit 1X dsDNA High Sensitivity DNA Assay kit (Cat. No. Q33230, Thermo Fisher Scientific, USA) and a Qubit Fluorometer (Thermo Fisher Scientific). The length of DNA library fragments was estimated using QIAxcel Advanced capillary gel electrophoresis with a QX DNA Fast Analysis kit (Cat. No. 929008, Qiagen, Germany). Sequencing was performed using an FCS flow cell on an MGI DNBSEQ-G50 platform (BGI, China) in the PE150 mode.

Bacterial genomes were assembled using SPAdes [8] in the “isolate” mode. To improve final assembly quality, raw reads were aligned to contigs using Bowtie2 [9], after which the alignment files were sorted and indexed using SAMtools [10] and transferred to Pilon [11] to correct assembly inaccuracies. Assembly quality was assessed using QUAST [12], and the completeness of the assembled genomes was evaluated using BUSCO [13]. The assembled genomic sequences were deposited in the NCBI database (BioProject: PRJNA1184111).

**Genome analysis**

Genome annotation and functional analysis were performed using the NCBI Prokaryotic Genome Annotation Pipeline [14] and the BV-BRC platform [15] that employs the RASTtk algorithm [16]. Comparative analysis of gene presence in propionibacterium was performed using the BV-BRC platform based on high-quality, open-access complete P. freudenreichii genome assemblies (n = 112).

**Identification of bacterial immune systems**

Bacterial immune systems were identified using the PADLOC software (v2.0.0) [17]. CRISPR repeats and spacers were identified using the CRISPR-Cas Finder tool (v4.3.2) [18], and Cas proteins were annotated using PADLOC. To identify potential targets, spacers were aligned to bacterial phage genomes using Bowtie2 v2.5.4 [19]. Nucleotide sequences of 575 phage genomes were obtained from the NCBI database (accessed July 4, 2025).

**Phylogenetic analysis**

Phylogenetic identification and determination of closely related strains were performed using tetranucleotide correlation analysis via the JSpeciesWS web service [20]. Average nucleotide identity (ANI) was compared using the OrthoANI algorithm [21].

## RESULTS

**General genomic characterization**

Whole-genome sequencing is considered the gold standard for genetic characterization of microorganisms. General characteristics of the genomic sequences of the five strains are presented in Table 1.

**Table 1:** Genomic characteristics of the study P. freudenreichii strains, based on whole-genome sequencing data

P. freudenreichii	FNCPS 2	FNCPS 3	FNCPS 4	FNCPS 6	FNCPS 828
Contigs	599	205	159	446	83
GC content	65.95	67.04	66.68	66.38	67.24
Contig L50	9	11	5	11	9
Genome length, bp	2806765	2894278	2649124	2734816	2579802
Contig N50	101.834	63836	169499	79634	93772
CDS	2.684	2.768	2.497	2.603	2.349
tRNA	48	173	44	58	45
Repeat regions	44	48	13	46	38
rRNA	3	3	4	3	4
Hypothetical proteins	935	933	790	845	670
Proteins with functional annotation	1.749	1.835	1.707	1.758	1.679

**Fig. 1:**
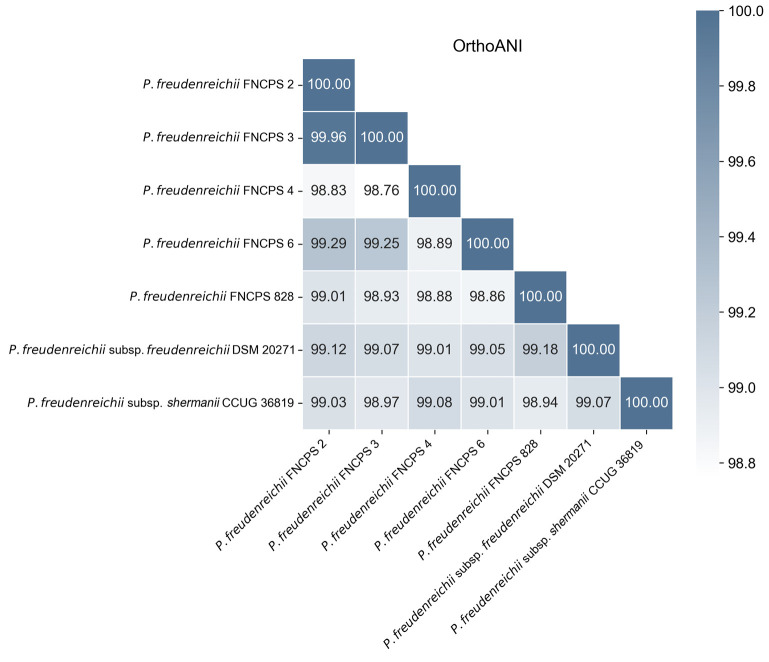
...

Tetracorrelation analysis revealed that the type strain P. freudenreichii subsp. shermanii CCUG 36819 had the closest similarity to the study strains P. freudenreichii FNCPS 828, 2, 6, and 4 (z-score: 0.99948, 0.96457, 0.97622, and 0.98812, respectively). P. freudenreichii subsp. freudenreichii DSM 20271 was closest to strain FNCPS 3 (z-score = 0.98812). Calculation of OrthoANI values among all the study strains and the reference genomes showed a high phylogenetic closeness (ANI > 98%), which indicates that they probably belong to the same clonal group. The results are presented as a pairwise similarity matrix (Fig. 1).

**Strain phenotyping**

Gas production. Gas production (CO_2_ production) is one of the key technological characteristics of propionic acid bacteria, which enables the formation of “eyes” in Emmental-type cheeses [22]. The main CO_2_ producer during ripening is P. freudenreichii that metabolizes lactic acid to form propionate, acetate, and CO_2_ .

**Fig. 2:**
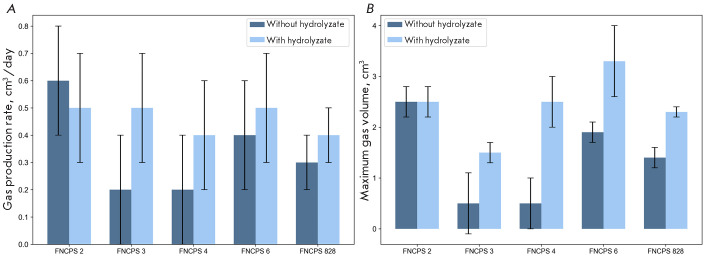
...

To assess gas-producing activity, we conducted experiments using two types of culture media: with and without milk hydrolyzate. The results are presented in Fig. 2A,B.

Strain P. freudenreichii FNCPS 2 growing on the medium without milk hydrolyzate was characterized by the highest rate and volume of gas production, whereas these indicators in strains FNCPS 3 and FNCPS 4 were low. On the medium with hydrolyzed milk, the differences among the strains decreased: activity of P. freudenreichii FNCPS 2 remained high, indicators of FNCPS 6 and FNCPS 828 significantly increased, while the volume of released gas in FNCPS 3 was low. Thus, strain FNCPS 2 is characterized by stable and high gas production, whereas FNCPS 3 exhibits low activity, regardless of culture conditions.

**Fig. 3:**
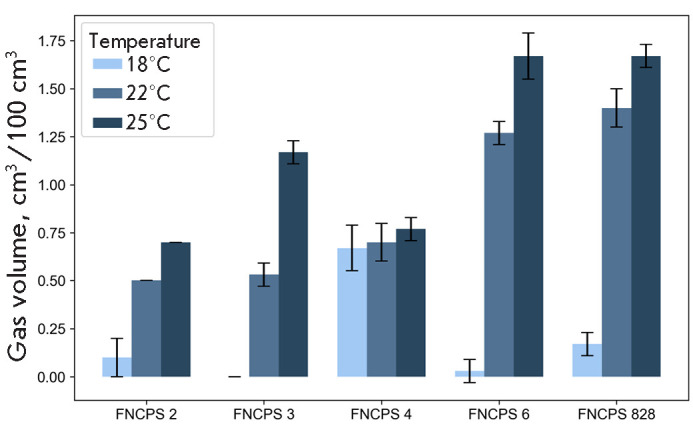
...

**Metabolic profiling of bacteria**

**Fig. 4:**
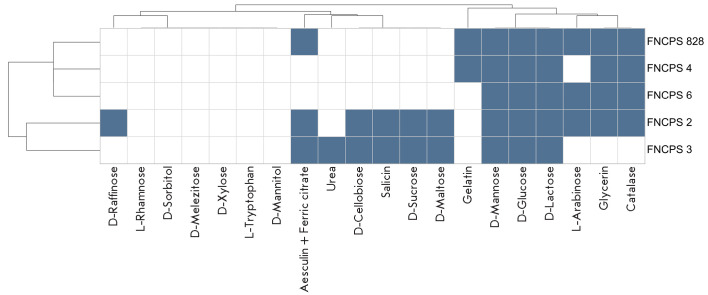
...

Metabolic profiling of the strains was performed using the BioMérieux system. The results are presented in Fig. 4.

According to the data, the only substrates metabolized by all the strains were D-mannose, D-glucose, and D-lactose. Strain FNCPS 3, unlike the other strains, was not able to utilize glycerol and lacked catalase activity. P. freudenreichii FNCPS 2 had the broadest metabolic profile (12 of 20 substrates tested). In addition, strains FNCPS 828 and FNCPS 4 were able to degrade gelatin, which was not typical of the other strains.

**Genome analysis**

**Fig. 5:**
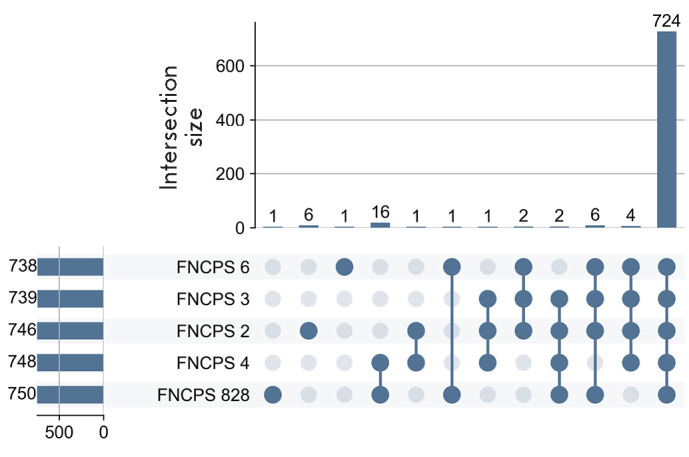
...

Analysis of the coding sequences at the role level of annotated biological gene functions revealed that all strains possessed a comparable number of unique functional groups (from 738 to 750), of which 724 were common to all (Fig. 5). The most related strains were FNCPS 4 and 828, which shared 16 unique functional gene groups.

Polymorphic variants of a number of genes in the study strains may play a key role in the cheese ripening process, influencing the organoleptic properties of products, the efficiency of metabolic pathways, and the overall nutritional value. For example, only strains P. freudenreichii FNCPS 4 and FNCPS 828 were found to contain a complete set of genes encoding the enzymes involved in 1,2-propanediol metabolism and components of the propanediol dehydratase complex (PduA, PduB, PduJ, PduK, PduN, PduU, and PduV genes), which indicates the ability of these strains to anaerobically convert propanediol into propanol and propionate [23].

Strains FNCPS 2, 3, and 6 contain aspartate racemase [EC 5.1.1.13] involved in the synthesis of D-aspartate, as well as the OppB gene encoding a component of an oligopeptide transporter that ensures the uptake of peptides from the environment - an important source of nitrogen in the fermentation matrix.

The only difference at the functional class level was the absence of genes belonging to the Nitrogen Metabolism class in strain FNCPS 828.

**Subspecies identification**

The P. freudenreichii species is traditionally divided into two subspecies: freudenreichii and shermanii. The main traits differentiating these subspecies are the ability to reduce nitrates and ferment lactose [4]. Usually, ssp. freudenreichii strains reduce nitrates but do not metabolize lactose, whereas ssp. shermanii are able to ferment lactose but not able to reduce nitrates [24].

**Fig. 6:**
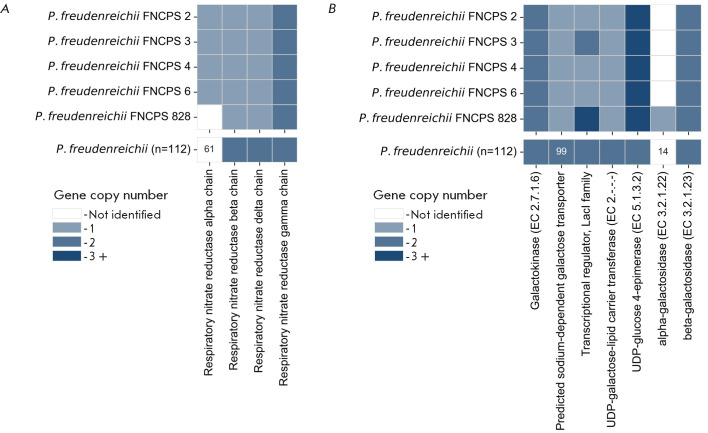
...

The key enzyme involved in nitrate reduction is the respiratory nitrate reductase complex [EC 1.7.99.4]. This enzyme functions as a final electron acceptor under anaerobic conditions, participating in energy generation. Genomic analysis revealed that strain FNCPS 828 lacked the narG gene encoding the respiratory nitrate reductase alpha chain that directly reduces nitrate to nitrite [25]. The lack of this gene was detected in 44 of 112 (39%) analyzed P. freudenreichii genomes (Fig. 6A). In this case, none of the narG gene-containing strains had been previously classified as a shermanii subspecies.

The only difference in the genetic profile of strain FNCPS 828 from the other study strains was the lack of the gene encoding α-galactosidase, an enzyme that breaks down α-D-galactooligosaccharides and polysaccharides, including melibiose, raffinose, stachyose, and verbascose. The α-galactosidase gene was detected in only 14% of P. freudenreichii genomes and was not identified in any of the typical representatives of ssp. freudenreichii (Fig. 6B).

**Characterization of bacterial defense systems**

General characterization. Bacteriophages represent a serious threat to propionic acid bacteria, because their infection decreases cellular metabolic activity, which is very important under production conditions [26]. During evolution, bacteria have developed a variety of defense systems against a bacteriophage infection.

**Fig. 7:**
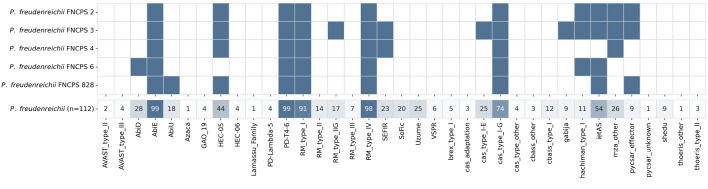
...

Analysis of 112 previously published P. freudenreichii genomes revealed that abortive infection (AbiE, PD-T4-6) and type I and IV restriction-modification (RM) systems were the most common bacterial immune systems, present in over 90% of the genomes. The type I-G CRISPR-Cas system was also quite common, being identified in 74% of the strains (83 of 112) (Fig. 7). All of these defense mechanisms were also identified in the five study strains.

In addition to the widespread systems, less well-studied anti-phage mechanisms were also found in the five analyzed genomes. For example, the HEC06 system, which uses nucleases to recognize and degrade modified phage DNA [27], was identified in all strains, except FNCPS 6. In the P. freudenreichii population, it is present in 44% of the genomes. The IetAS system, which is characteristic of 54% of strains in this species, is present in all study genomes, except FNCPS 3. Although its mechanism of action has not yet been fully elucidated, it is believed to function synergistically with other defense systems [28].

Interestingly, despite the relatively high detection frequency of systems such as SoFic (20%) and Uzume (25%) in P. freudenreichii strains, they were absent in all five study genomes. However, systems less common in the population, Hachiman I (11%) and Pycsar (9%), were identified in three of the five strains.

**CRISPR-Cas**

CRISPR-Cas is one of the best-known adaptive defense systems, which provides immunity to previously encountered phages by integrating fragments of their DNA into the bacterial genome [29]. The presence and composition of CRISPR-Cas systems in the five study P. freudenreichii strains were analyzed (Table 2).

**Table 2:** Characterization of CRISPR-Cas systems in the P. freudenreichii strains studied

P. freudenreichii strain	CRISPR‒Cas system, type	Cas proteins, number	Unique spacers, number	Unique CRISPRs, number
FNCPS 2	I‒G	6	44	2
FNCPS 3	I‒G, I‒E	14	170	5
FNCPS 4	I‒G	6	13	3
FNCPS 6	I‒G	6	62	4
FNCPS 828	I‒G	5	37	2

Strains FNCPS 2, FNCPS 4, and FNCPS 6 were found to possess a complete type I-G system including all the necessary proteins: Cas1, Cas2, Cas3, Cas56, Cas7, and Cas8, which indicates its potential functionality. Strain FNCPS 3 contains two CRISPR-Cas systems: complete I-G and I-E clusters (Cas1, Cas2, Cas3, Cas5, Cas6, Cas7, Cas8, and Cas11), which may provide increased resistance to foreign DNA.

Strain FNCPS 828 was found to possess an incomplete I-G CRISPR-Cas system: it lacked the Cas2 protein involved in inserting new spacers into the CRISPR array. However, other key components were present.

**Fig. 8:**
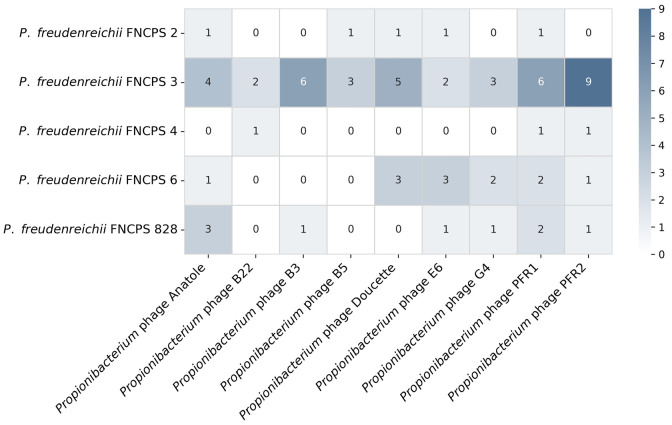
...

A total of 326 unique spacers were identified in the five P. freudenreichii strains. To search for potential targets of CRISPR-Cas systems, we aligned the identified spacers with the previously published genomes of 575 bacteriophages infecting bacteria used in the dairy industry. The analysis revealed matches of 69 spacers (21%) with the genomes of nine different phages, all of which infect propionibacterium (Fig. 8).

## DISCUSSION

This paper presents the results of an analysis of five phylogenetically related P. freudenreichii strains with significant phenotypic differences. Despite a high degree of genomic identity (orthoANI > 99.9), strains P. freudenreichii FNCPS 2 and FNCPS 3 differed significantly in a number of phenotypic traits. P. freudenreichii FNCPS 2 exhibited stable and high gas-producing activity, both in standard medium and in medium with milk hydrolyzate, whereas FNCPS 3 produced gas only in the presence of hydrolyzate. Both strains were grouped into a single cluster based on their metabolic profiles, indicating adaptation to the environment of their common origin. However, FNCPS 3 did not utilize L-arabinose, D-raffinose, and glycerol, compounds potentially present in the dairy medium, whereas FNCPS 2 metabolized the largest number of substrates, which is consistent with its pronounced gas-forming activity. Previously, the intensity of gas production in P. freudenreichii was shown to be directly related to the availability of metabolites, primarily lactate: upon nutrient depletion, the fermentation level and CO_2_ release decreased [30]. Also, the use of carbon substrates, such as whey lactose and glycerol, affects fermentation and gas production in P. freudenreichii ssp. shermanii, confirming the dependence of this process on the type and diversity of nutrient sources [31, 32]. Thus, differences in the metabolism of easily digestible carbon sources likely explain the observed differences in gas production among strains [33].

Recent studies have demonstrated that the traditional division of P. freudenreichii into the subspecies freudenreichii and shermanii, based on the ability to ferment lactose and reduce nitrates, does not reflect the actual genetic and phenotypic diversity of this species. Strains with various combinations of these traits have been reported [24, 34], and phylogenetic analysis using MLST has not revealed a clear clustering consistent with the existing subspecies classification [34]. A recent phenotype-based reclassification showed that more than 45% of the strains examined could not be assigned to any of the subspecies [24]. In addition, some strains were probably misclassified as non-nitrate-reducing due to insufficient incubation time [24]. In this regard, the development of genetic markers to correctly distinguish subspecies and predict phenotypes is becoming topical.

Strain FNCPS 828 demonstrated high phylogenetic closeness to type strain P. freudenreichii subsp. shermanii CCUG 36819 (ESK = 0.99948) and was capable of lactose metabolism, but lacked the full set of genes required for nitrate reduction. The only variable gene associated with this ability in P. freudenreichii strains was narG encoding the respiratory nitrate reductase alpha subunit, which makes it a potential marker for subspecies identification.

To date, a limited number of studies have been devoted to the defense systems of P. freudenreichii. The most common defense mechanism in bacteria is the restriction-modification system [35] also identified in P. freudenreichii [36]. As previously reported, the most common CRISPR-Cas system in these bacteria is type I-G, although type I-E is also present [37]. Our analysis of 112 P. freudenreichii strains confirmed the predominance of the I-G system, whereas the I-E system was present in approximately 25% of the strains. In addition, AbiE, PD-T4-6, and type I and IV restriction-modification systems were found in more than 90% of the strains analyzed.

All the five study strains contained the type I-G CRISPR-Cas system. In strain FNCPS 828, this system was incomplete and lacked the gene encoding the Cas2 protein. Strain FNCPS 3, in addition to the type I-G system, also possessed an additional type I-E CRISPR-Cas system. This strain was also characterized by the highest number of spacer sequences, including the highest number of spacers whose targets matched previously reported propionibacterium phages. Previously, P. freudenreichii strains were reported to contain spacers to phages B22, Anatole, E1, Doucette, E6, G4, and B3 [38]. In our study, only strain FNCPS 3 had spacers to all previously described phages, except E1, and also contained additional spacers to phages B5, PFR1, and PFR2, which reflects significant viral pressure during the co-evolution of the strain with bacteriophages. In addition to CRISPR- Cas systems, all strains were found to possess the most common defense mechanisms in P. freudenreichii, as well as the less studied HEC-06 and ietAS complexes, which indicates a layered antiviral defense system in members of this species.

## CONCLUSIONS

In this study, we analyzed both the common traits and intraspecific diversity of P. freudenreichii strains, which has direct implications for the dairy industry. Differences in gas-producing activity, the range of metabolized substrates, and bacterial defense systems reflect the adaptation of strains to various technological conditions and underscore the need for their targeted selection to optimize starter cultures. The identification of the narG gene as a potential marker for nitrate reduction and the description of defense system diversity, including CRISPR-Cas, open up prospects for more accurate strain typing and prediction of their technological properties. These findings provide the basis for the development of starter cultures with increased stability, predictable characteristics, and resistance to bacteriophages, which ultimately facilitates the generation of more reliable and functional industrial cultures adapted to the profile of specific fermentation processes.
